# The Potential Association Between Betel Nut Consumption and Apical Periodontitis: A Multicenter Cross‐Sectional Study

**DOI:** 10.1155/ijod/2691950

**Published:** 2026-06-19

**Authors:** Tousif Iqbal Nathani, Vaibhav Kumar, Satvinder Singh, Shishir Singh, Marc Encinas, Fernando Durán-Sindreu, Juan Gonzalo Olivieri

**Affiliations:** ^1^ Department of Endodontics, International University of Catalonia (UIC), Barcelona, Spain, uic.es; ^2^ Department of Public Health Dentistry, Dr. GD Pol Foundation YMT Dental College and Hospital, Navi Mumbai, India; ^3^ Department of Oral Medicine and Radiology, Indira Gandhi Govt. Dental College and Hospital, Amphala, Jammu, Jammu and Kashmir, India, iggdcj.edu.in; ^4^ Department of Conservative Dentistry and Endodontics, Terna Dental College, Nerul, Navi Mumbai, India, ternadental.org

**Keywords:** areca, betel nut, endodontics, epidemiologic studies, oral health, periapical periodontitis

## Abstract

**Introduction:**

Chewing betel nut (BN) is a well‐established risk factor for several oral conditions, including periodontal diseases, but direct evidence linking BN chewing to pulpal and periapical (PA) pathology remains scarce. This study aimed to assess the association between BN chewing and apical periodontitis (AP).

**Materials and Methods:**

A cross‐sectional observational study was conducted at two dental institutions in India from January 2021 to March 2023. A total of 222 participants were enrolled, comprising 113 habitual BN chewers (≥1 packet/day for ≥1 year) and 109 nonchewers with no history of tobacco use. Demographic, clinical, and radiographic data were collected through interviews, clinical, and radiographic examinations. Two calibrated endodontists evaluated radiographs to identify PA radiolucencies. Statistical analysis included chi‐square tests, Mann–Whitney *U* tests, and Poisson log‐linear regression models to assess the association between BN chewing and the number of AP lesions (*α* = 0.05).

**Results:**

BN chewers presented a higher number of AP lesions than nonchewers (*p* < 0.05). Poisson regression analysis showed that BN chewing was significantly associated with a higher number of AP lesions in both unadjusted (incidence rate ratio [IRR] = 1.504, *p*  < 0.001) and fully adjusted models (IRR = 1.290, *p* = 0.034). Further analysis revealed a dose‐dependent relationship, with each additional packet of BN chewed per day increasing the likelihood of AP lesions by ~11% and each additional year of BN use by around 3%–4%. No differences in the prevalence of carious lesions were observed between groups (*p* > 0.05).

**Conclusion:**

BN chewing was independently associated with a higher prevalence of radiographic AP. Longer chewing duration was associated more strongly with PA lesion counts than chewing frequency. Given the cross‐sectional design, these findings indicate association rather than causation, and longitudinal studies accounting for additional confounders are needed.

## 1. Introduction

Betel or areca nut (BN) seed, derived from the *Areca catechu* palm, is one of the most widely consumed psychoactive substances globally [1]. It is listed as a Class I carcinogen by the International Agency for Research on Cancer (IARC) [2]. Globally, the consumption of BN is estimated to occur in around 10% of the population [3], with South and Southeast Asia being the regions with the highest prevalence. In India, approximately one‐fourth of the population consumes BN, with more than 40% of the population in some states [4]. The north‐east and central regions of India have the highest BN use compared with the southern regions [5]. Factors influencing its usage include sex, age, marital status, and education level [4, 5].

The contents of BN quid primarily include betel leaf, areca nut, catechu, slaked lime, and sometimes tobacco. In Southeast Asia, this mixture is referred to as paan, and its ingredients can vary depending on the preparation method and regional practices [6]. Typically, the term “paan masala” describes a mixture that does not contain tobacco, while “gutka” relates to the tobacco‐containing variant of paan masala [7]. A key chemical component of BN quid is arecoline, an alkaloid classified as a carcinogen, which has been strongly associated with oral cancers and other severe health conditions [8]. In addition to its carcinogenic effects, BN use has also been linked to a higher risk of chronic systemic diseases such as diabetes mellitus (DM), cardiovascular diseases, including hypertension (HT) and ischemic heart diseases [9], and kidney diseases [10], further underscoring its significant adverse health impacts. Beyond these local effects, oral diseases such as periodontitis, apical periodontitis (AP), peri‑implantitis, caries, and oral potentially malignant disorders are increasingly recognized as being closely associated with metabolic syndrome, suggesting shared inflammatory and cardiometabolic pathways [11]. BN chewing is also associated with metabolic disturbances and components of metabolic syndrome, which may further amplify systemic and oral disease risk in this population [12, 13].

Habitual BN chewing leads to several oral pathologies, including oral submucous fibrosis (OSF), oral cancer, and precancerous lesions such as leukoplakia [2]. The carcinogenic compounds found in BN are linked to mutations in oral mucosal cells, making regular users particularly vulnerable to malignant transformations [14]. Additionally, this habit has been shown to significantly increase the risk of periodontal diseases, resulting in up to 13 times more gingival bleeding, clinical attachment loss, and marginal bone loss [15]. Consuming BN at elevated levels of intensity and frequency can initiate a cascade of dental pathologies. The progression of oral deterioration typically begins with tooth wear, dental plaque, and calculus accumulation, eventually leading to more severe conditions, such as periodontitis, alveolar bone loss, and, in extreme cases, tooth loss [16]. Attrition and abrasion of the occlusal surfaces and an increased number of tooth fractures have also been observed as common in habitual BN chewers [17]. The extent of tooth wear is influenced by various factors, including BN’s hardness, the habit’s duration, and the frequency of chewing [18]. Tooth enamel loss can result in dentin exposure, potentially accelerating wear and leading to dentinal hypersensitivity, pulpitis, pulp necrosis, and, ultimately, periapical (PA) disease [19, 20]. Prolonged BN chewing has also been associated with severe occlusal wear, marginal bone loss, and even tooth loss [21].

AP is an inflammatory condition that occurs around the apex of a tooth root, primarily caused by a bacterial infection of the pulp [22]. A recent systematic review found that more than half of the world’s population who attended clinical consultation presented at least one tooth with a radiolucency compatible with AP [23]. Several factors seemed to increase the rate of presenting AP, including having systemic conditions, tobacco smoking, and living in developing nations [23]. Similarly, several studies have found a higher incidence of AP in smokers compared to nonsmokers [24, 25]. In addition, smoking tobacco results in a higher risk of presenting dental carious lesions and thicker biofilms [26]. Tobacco contains both stable and unstable free radicals, as well as the formation of reactive oxygen species, which can lead to oxidative damage within biological systems and potentially contribute to malignancy [27]. Moreover, tobacco use has been associated with decreased vascularization and a diminished immune response, characterized by the suppression of leukocytes, macrophages, and T‐cell lymphocytes, as well as increased cytokine production and the release of inflammatory mediators, such as tumor necrosis factor‐alpha (TNF‐α) [28]. These findings are considered to be one of the reasons why smokers do not adequately mitigate damage from bacterial infections, particularly in the pulp and surrounding tissues [29]. Similar findings have been reported among individuals who consume BN concerning their immune system functionality with a reduction in immune activity, accompanied by diminished T‐cell production, increased cytokine production (TNF‐α), interleukin‐8 (IL‐8), and heightened cell proliferation [30].

Given that AP is primarily caused by pulpal infection, any potential link between BN chewing and AP is likely indirect and should be considered exploratory. BN chewing may contribute through factors such as occlusal wear, microcracks or fractures, restoration breakdown, and altered inflammatory responses, which could increase susceptibility to pulpal infection and subsequent apical pathology. Therefore, the objective of the present study was to compare the prevalence of AP among BN chewers and nonchewers. The working hypothesis posited that BN chewers exhibit a higher prevalence of AP than their nonchewing counterparts.

## 2. Materials and Methods

### 2.1. Study Design and Setting

The present study was an observational, cross‐sectional, multicenter study conducted at the Indira Gandhi Government Dental College (Jammu, India) and Terna Dental College and Hospital (Navi Mumbai, India). The guidelines for observational studies of the Strengthening the Reporting of Observational Studies in Epidemiology (STROBE) were followed. The ethics committees of the respective colleges approved the study protocol (study codes: IEC/GDC32/20 and TDC/EC/17/2021).

The study was reported in accordance with the STROBE checklist, which has been provided as Supporting Information.

### 2.2. Case Selection

Patients who visited the outpatient department of the dental faculty for a new patient or routine examination at both centers were included in the study between January 2021 and March 2023. The following inclusion and exclusion criteria were applied until each subgroup (center 1 BN chewers, center 1 nonchewers, center 2 BN chewers, and center 2 nonchewers) was completed.

### 2.3. Inclusion and Exclusion Criteria

The inclusion criteria were patients aged over 18 years, willing to participate, and provided written informed consent, who were regular BN consumers (≥1 packet daily for at least 1 year), or those who had never used any form of BN or tobacco products. The exclusion criteria were as follows: (1) patients who used BN with tobacco/cigarette smoking; (2) patients with terminal illness; (3) pregnant or lactating women; and (4) American Society of Anesthesiologists (ASA) III, IV, or V patients. The ASA physical status classification ranges from ASA I (healthy) and ASA II (mild systemic disease) to ASA III–V (severe systemic disease to moribund); to reduce medical confounding, ASA III–V were excluded.

### 2.4. Variables and Measurements

#### 2.4.1. Demographic and Clinical Data

• Demographic data (age, sex, level of education, and number of children).
• Systemic diseases (only included hyper/hypothyroidism, diabetes, and/or HT).
• Toxic habits: BN usage (number of packets and years of use) and alcohol (occasionally/daily).

#### 2.4.2. Intraoral and Radiographic Examination

• Intraoral examination: carious lesions (International Caries Detection and Assessment System: ICDAS scoring criteria) [31], number and adequacy of dental restorations (inadequate restorations were identified by clinical or radiographic observations of open margins, overhangs, secondary decay, or exposed restorations).
• Presence and number of PA lesions.

### 2.5. Sample Size Calculation

A sample size calculator (GRANMO 7.12, IMIM, Barcelona, Spain) was used to determine the required sample size based on the prevalence of AP reported by Balto et al. [32]. With an assumed alpha risk of 0.05 and 0.8 power for a bilateral contrast and accounting for an anticipated 10% loss due to possible inadequacies in data registration, the total estimated sample size was 50 subjects per subgroup (200 patients in total).

### 2.6. Procedure

A standardized form was employed to document the demographics, habits, and medical history details of patients attending the clinics. The patient’s intraoral examination was conducted by a postgraduate (PG) student from the respective centers’ oral medicine and radiology departments (Indira Gandhi Govt. Medical College, Jammu and Terna Dental College, Navi Mumbai). Data related to oral examination findings, including the number of teeth, the presence of carious lesions, and the presence and quality of dental restorations, were recorded by a previously calibrated PG student.

Orthopantomography (OPG) x‐rays were acquired using a panoramic dental imaging unit (Planmeca ProMax 2D) with the following standardized parameters: 66 kVp, 8 mA, and 16 s exposure. Two previously calibrated endodontists with over 5 years of experience reviewed the OPGs and recorded the presence of PA radiolucency compatible with PA lesions for all teeth.

PA radiolucency was defined as a radiolucent area associated with the root apex that exceeded the normal periodontal ligament space and/or showed a loss of lamina dura continuity, compatible with AP [33]. Only the number of teeth presenting such apical radiolucencies was recorded for analysis, without differentiation by tooth type. Calibration was performed before study commencement using a set of training OPGs, with disagreements resolved by consensus. Interexaminer agreement was high (Cohen’s kappa = 0.85).

### 2.7. Data and Statistical Analysis

The data were registered in a Microsoft Excel 2010 worksheet (Microsoft Ltd., Washington, USA). SPSS software v26.0 was used for statistical analysis. The data were subjected to normality assessment using the Kolmogorov–Smirnov test. Cohen’s kappa was used to assess interexaminer reliability. An independent *t*‐test was used to compare the age. Chi‐square or Fisher exact tests were used for group comparisons. The Mann–Whitney *U* test was used to compare the ICDAS index and the number of teeth exhibiting PA lesions among the two populations. The Spearman rank correlation test was used to assess the correlation between inadequate restorations and PA lesions. Poisson log‐linear regression models were applied to evaluate the association between BN chewing and the number of PA lesions. Both unadjusted and adjusted models were constructed. Individual covariates were included in the adjusted models to control for potential confounding. Study center (Jammu vs. Mumbai) was included as a covariate in multivariable models to account for center‐level differences. The fully adjusted model included all covariates that showed statistical significance in the individual models. The results were reported as incidence rate ratios (IRRs) with 95% confidence intervals (CIs), and a *p*‐value of less than 0.05 was considered statistically significant.

## 3. Results

The study’s final sample consisted of 222 patients. One hundred thirteen participants were included in the BN group (54 from Indira Gandhi Government Dental College in Jammu and 59 from Terna Dental College and Hospital in Navi Mumbai) and 109 participants in the non‐BN group (50 and 59 of the two different centers, respectively). Sample distribution is displayed in Table 1.

**Table 1:** Demographic characteristics of the study participants, Betel nut chewers and non‐chewers.

Variable	Category	*N*	BN chewing		*p*‐Value
			Yes	No	
Age	Mean ± SD	222	43.26 ± 11.83	35.28 ± 11.75	<0.001 ^∗^
Sex	Male	135	83 (73.5%)	52 (47.7%)	<0.001 ^∗^
	Female	87	30 (26.5%)	57 (52.3%)	
Marital status	Married	171	98 (86.7%)	73 (67%)	0.001 ^∗^
	Single	51	15 (13.3%)	36 (33%)	
Level of education	No education	33	16 (14.2%)	17 (15.6%)	<0.001 ^∗^
	Till 12^th^	105	73 (64.6%)	32 (29.4%)	
	UG	65	16 (14.2%)	49 (45%)	
	PG	19	8 (7.1%)	11 (10.1%)	
Number of children	0	36	11 (10.4%)	25 (28.1%)	0.006 ^∗^
	1	55	40 (37.7%)	15 (16.9%)	
	2	78	39 (36.8%)	39 (36.8%)	
	3	24	12 (11.3%)	12 (11.3%)	
	4	6	3 (2.8%)	3 (2.8%)	
	5	2	1 (0.9%)	1 (1.1%)	
Medical history	None	162	78 (69%)	84 (77.1%)	0.596
	Diabetes	20	13 (11.5%)	7 (6.4%)	
	Hypertension	27	15 (13.3%)	12 (11%)	
	DM + HT	10	6 (5.3%)	4 (3.7%)	
	Hyperthyroidism	1	0	1 (0.9%)	
	Hypothyroidism	2	1 (0.9%)	1 (0.9%)	
*Oral habits*					
Dental visits	No visit	31	19 (16.8%)	12 (11%)	0.324
	1 visit	177	85 (75.2%)	92 (84.4%)	
	2 visits	13	8 (7.1%)	5 (4.6%)	
	>2 visits	1	1 (0.9%)	0	
Toothbrushing	0 times	2	0	2 (1.8%)	0.209
	Once	209	109 (96.5%)	100 (91.7%)	
	Twice	11	4 (3.5%)	7 (6.4%)	
Flossing	No	210	113 (100%)	97 (89%)	<0.001 ^∗^
	Yes	12	0	12 (11%)	
*Toxic habits*					
Alcohol consumption	Yes		30 (26.5%)	11 (10.1%)	0.002 ^∗^
	No		83 (73.5%)	98 (89.9%)	
BN chewing	No. of packets	Mean ± SD	2.09 ± 1.25	—	—
	Duration	Mean ± SD	14.14 ± 9.38	—	
*Carious lesion status*					
ICDAS	ICDAS (0–2)	Mean ± SD	2.43 ± 1.29	2.77 ± 0.89	0.155
	ICDAS (3–4)	Mean ± SD	2.44 ± 1.27	2.74 ± 1.24	0.098
	ICDAS (5–6)	Mean ± SD	3.48 ± 2.68	3.33 ± 2.53	0.707
*Apical Periodontitis*					
PA lesions		Mean ± SD	1.85 ± 1.93	1.23 ± 1.57	0.009 ^∗^

*Note:* Independent *t*‐test, chi‐square test, and Fisher exact test.

Abbreviations: BN, betel nut; DM, diabetes mellitus; HT, hypertension; ICDAS, International Caries Detection and Assessment System; PA, periapical; PG, postgraduate; SD, standard deviation; UG, undergraduate.

^∗^Indicates a significant difference at *p* ≤ 0.05.

A higher proportion of males were BN chewers (73.5%) compared to nonchewers (47.7%) (*p*  < 0.001). BN chewers were significantly older (43.26 ± 11.83 years) than nonchewers (35.28 ± 11.75 years) (*p* < 0.05). A higher number of BN chewers had a lower level of education (*p* < 0.05) and had a comparatively higher number of children (*p* < 0.05). The intake of alcohol was also seen to be significantly higher in BN chewers compared to nonchewers (*p* < 0.05). There was no significant difference in the number of dental visits or brushing habits between the two groups (*p* > 0.05). However, the dental flossing habit was more prevalent among nonchewers (*p* < 0.05). The comparison of the ICDAS index between BN chewers and nonchewers showed no differences across all categories, indicating a similar number of carious lesions (*p* > 0.05) (Table 1).

A higher number of PA lesions were observed in the BN group (1.85 ± 1.93) than in nonchewers (1.23 ± 1.57) (*p* < 0.05) (Table 1). In addition, a significant correlation was found between inadequate crowns and PA lesions in all participants (*p* < 0.05). Interexaminer reliability was 0.85.

Poisson log‐linear regression analysis revealed that BN chewing was significantly associated with a higher number of PA lesions. In the unadjusted model, BN chewers had a 50.4% higher count of PA lesions compared to nonchewers (Exp (B) = 1.504, *p*  < 0.001). This association remained significant when adjusted for individual variables such as sex (Exp (B) = 1.582, *p*  < 0.001), systemic disease (Exp (B) = 1.451, *p* = 0.001), dental visits per year (Exp (B) = 1.499, *p*  < 0.001), brushing habits (Exp (B) = 1.490, *p*  < 0.001), alcohol use (Exp (B) = 1.472, *p* = 0.001), decayed teeth (Exp (B) = 1.544, *p*  < 0.001), marital status (Exp (B) = 1.348, *p* = 0.008), education level (Exp (B) = 1.382, *p* = 0.004), and flossing habits (Exp (B) = 1.402, *p* = 0.003). The association remained significant in the fully adjusted model incorporating all significant covariates, where BN chewers still exhibited a 29% higher expected number of PA lesions compared to nonchewers (Exp (B) = 1.290, *p* = 0.034). These findings suggest that BN chewing is independently associated with an increased number of PA lesions, even after adjusting for potential clinical and demographic confounders (Table 2).

**Table 2:** Association between betel nut chewing and periapical lesions.

Covariates included	*β*	Exp (B)	95% CI for Exp (B)	*p*‐Value
Unadjusted model	0.408	1.504	1.211–1.869	<0.001 ^∗^
Age	0.213	1.237	0.985–1.554	0.067
Sex	0.458	1.582	1.263–1.980	<0.001 ^∗^
Systemic disease	0.372	1.451	1.167–1.804	0.001 ^∗^
Dental visits per year	0.405	1.499	1.206–1.862	<0.001 ^∗^
Tooth brushing habits	0.399	1.49	1.197–1.854	<0.001 ^∗^
Alcohol use	0.387	1.472	1.179–1.837	0.001 ^∗^
Decayed teeth	0.434	1.544	1.243–1.918	<0.001 ^∗^
Marital status	0.299	1.348	1.081–1.682	0.008 ^∗^
Level of education	0.323	1.382	1.108–1.722	0.004 ^∗^
Flossing habits	0.338	1.402	1.125–1.746	0.003 ^∗^
All significant covariates together	0.255	1.29	1.019–1.633	0.034 ^∗^

*Note:* Exp (B), exponentiated regression coefficient.

^∗^Poisson’s log‐linear regression.

Dose‐response analyses by frequency of BN chewing are presented in Table 3. In unadjusted models, a higher reported frequency was associated with a higher expected count of PA lesions. After full adjustment, the association of frequency attenuated and did not remain statistically significant (*p* = 0.066), although the direction of the effect remained positive.

**Table 3:** Association between the frequency of betel nut chewing (number of packets per day) with periapical lesions.

Covariates included	*β*	Exp (B)	95% CI for Exp (B)	*p*‐Value
Unadjusted model	0.106	1.112	1.011–1.224	0.028 ^∗^
Age	0.084	1.088	0.983–1.205	0.103
Sex	0.108	1.114	1.012–1.227	0.028 ^∗^
Systemic disease	0.104	1.109	1.005–1.222	0.039 ^∗^
Dental visits/year	0.097	1.102	1.002–1.213	0.046 ^∗^
Toothbrushing	0.107	1.113	1.012–1.223	0.028 ^∗^
Alcohol	0.098	1.103	1.001–1.216	0.048 ^∗^
Decayed teeth	0.106	1.112	1.011–1.224	0.028 ^∗^
All significant covariates	0.104	1.110	0.993–1.241	0.066

*Note*: Exp (B), exponentiated regression coefficient.

^∗^Poisson’s log‐linear regression.

Dose‐response analyses by duration of BN chewing are presented in Table 4. Duration showed a consistent positive association with the number of PA lesions and remained statistically significant after full adjustment (*p* = 0.001).

**Table 4:** Association between duration of betel nut chewing (in years) with periapical lesions.

Covariates included	*β*	Exp (B)	95% CI for Exp (B)	*p*‐Value
Unadjusted model	0.032	1.032	1.019–1.046	<0.001 ^∗^
Age	0.041	1.042	1.019–1.065	<0.001 ^∗^
Sex	0.032	1.032	1.019–1.046	<0.001 ^∗^
Systemic disease	0.046	1.047	1.029–1.065	<0.001 ^∗^
Dental visits/year	0.035	1.036	1.022–1.050	<0.001 ^∗^
Toothbrushing	0.024	1.024	1.010–1.039	<0.001 ^∗^
Alcohol	0.033	1.033	1.019–1.047	<0.001 ^∗^
Decayed teeth	0.021	1.021	1.007–1.036	0.003 ^∗^
All significant covariates together	0.045	1.046	1.019–1.075	0.001 ^∗^

*Note*: Exp (B), exponentiated regression coefficient.

^∗^Poisson’s log‐linear regression.

Subjects in Jammu reported a higher education level, more frequent brushing habits, and a higher number of children per individual compared to those in Mumbai (*p* < 0.05). Jammu subjects also presented with lower ICDAS values (*p* < 0.05). There were no significant differences in the mean number of PA lesions between centers (*p* = 0.274) (Table 5). Among BN chewers, there were no significant center differences in the number of packets consumed per day or duration of chewing (*p*  > 0.05) (Table 6).

**Table 5:** Demographic and clinical characteristics of the study participants displayed by center (Jammu and Mumbai).

Variable	Category	*N*	Region		*p*‐Value
			Jammu	Mumbai	
Age	Years ± SD	222	38.24 ± 12.42	40.51 ± 12.46	0.177
Sex	Male	135	84 (80.8%)	51 (43.2%)	<0.001 ^∗^
	Female	87	20 (19.2%)	67 (56.8%)	
Marital status	Married	171	75 (72.1%)	96 (81.4%)	0.112
	Single	51	29 (27.9%)	22 (18.6%)	
Level of education	No education	33	19 (18.3%)	14 (11.9%)	0.004 ^∗^
	Till 12^th^	105	37 (35.6%)	68 (57.6%)	
	UG	65	34 (32.7%)	31 (26.3%)	
	PG	19	14 (13.5%)	5 (4.2%)	
Number of children	0	63	34 (32.7%)	29 (24.6%)	0.029 ^∗^
	1	55	17 (16.3%)	38 (32.2%)	
	2	76	34 (32.7%)	42 (35.6%)	
	>3	28	19 (18.5%)	9 (7.6%)	
Medical history	Healthy	162	83 (79.8%)	79 (66.9%)	0.016 ^∗^
	Diabetes	20	3 (2.9%)	17 (14.4%)	
	Hypertension	27	10 (9.6%)	17 (14.4%)	
	DM + HTN	10	5 (4.8%)	5 (4.2%)	
	Hyperthyroidism	1	1 (1%)	0	
	Hypothyroidism	2	2 (1.9%)	0	
*Oral habits*					
Dental visits	No visit	31	28 (26.9%)	3 (2.5%)	<0.001 ^∗^
	1 visit	177	65 (62.5%)	112 (94.9%)	
	2 visits	13	10 (9.6%)	3 (2.5%)	
	>2 visits	1	1 (1%)	0	
Toothbrushing	No	2	1 (1%)	1 (0.8%)	0.011 ^∗^
	Once/day	209	93 (89.4%)	116 (98.3%)	
	Twice/day	11	10 (9.6%)	1 (0.8%)	
Flossing	No	210	95 (91.3%)	115 (97.5%)	0.071
	Yes	12	9 (8.7%)	3 (2.5%)	
Toxic habits					
Betel nut habit	Chewers	113	54 (51.90%)	59 (50%)	0.789
	Nonchewers	109	50 (48.10%)	59 (50%)	
*Caries status*					
ICDAS	ICDAS (0–2)	Mean ± SD	2.37 ± 1.35	2.81 ± 0.82	0.008 ^∗^
	ICDAS (3–4)	Mean ± SD	2.23 ± 1.37	2.91 ± 1.06	<0.001 ^∗^
	ICDAS (5–6)	Mean ± SD	2.98 ± 0.36	3.78 ± 2.76	0.026 ^∗^
Apical periodontitis					
PA lesions		Mean ± SD	1.49 ± 1.95	1.59 ± 1.63	0.274

*Note:* Independent *t*‐test, chi‐square test, and Fisher exact test.

Abbreviations: DM, diabetes mellitus; HT, hypertension; ICDAS, International Caries Detection and Assessment System; PA, periapical; PG, postgraduate; SD, standard deviation; UG, undergraduate.

^∗^Indicates a significant difference at *p* ≤ 0.05.

**Table 6:** Betel nut chewing characteristics among BN chewers by center (mean ± SD).

Variable	Jammu	Mumbai	*p*‐Value
Number of packets per day	1.21 ± 1.67	0.93 ± 1.04	0.648
Duration of BN chewing (years)	7.76 ± 10.33	6.70 ± 9.19	0.576

*Note:* Values are reported as mean ± standard deviation among BN chewers only. *p*‐Values were calculated using the Mann–Whitney *U* test. Statistical significance was set at *p* ≤ 0.05.

Abbreviation: BN, betel nut.

## 4. Discussion

BN chewing has deleterious effects on the oral cavity and is associated with several oral diseases. Individuals have presented a higher prevalence of precancerous lesions like OSF, oral cancer, and also periodontal and other oral diseases and conditions [2, 15]. The present study was an observational cross‐sectional study in which the presence of AP in BN chewers was compared to that of nonchewers attending two different dental university clinics located in separate geographical regions of India (Jammu and Navi Mumbai).

Chronic BN chewers frequently experience significant attrition of tooth cusps, leading to the loss of occlusal pits and fissures. This has been thought to reduce the stagnation areas where decay usually begins [18]. Continuous BN chewing induces high salivary flow, which, combined with slaked lime, raises the oral pH and increases the formation of sclerosed dentin, further reducing the risk of caries [18]. BN chewers often have betel stains coating the tooth surfaces, and the tannin content has demonstrated to have antimicrobial properties, which may contribute to reduced cariogenic bacterial activity [34]. However, in the current study, there was no difference in the number of carious lesions between the BN chewers and nonchewers (*p* > 0.05). Whether BN chewing has a protective effect or not is still unknown, as well as the reason for discrepancies between studies. However, it can be attributed that the development of carious lesions is multifactorial, including oral hygiene habits, diet, socioeconomic status, level of academic education, or the number of dental visits, which can make it challenging to analyze homogeneous samples [35]. In the current study, a significant number of BN chewers had lower levels of academic education and inadequate oral hygiene habits, including brushing their teeth only once a day and not using floss as an aid in their oral hygiene routine (*p* < 0.05).

Tooth wear is a typical finding in BN chewers. Molar teeth, premolars, and canines often present an absence of an entire cuspal shape, while incisors become noticeably shortened [18]. The severity of the abrasion is directly dependent on the frequency and duration of the habit [12]. Extensive tooth wear can eventually lead to pulp exposure, which may result in pulpal and PA diseases [36]. However, there is a lack of data on the association between BN chewing and PA diseases. The present study showed that BN chewers had a higher prevalence of AP compared to nonchewers (*p* < 0.05). Furthermore, Poisson regression analysis confirmed that BN chewing was significantly associated with an increased number of AP lesions. The unadjusted model showed a 50.4% higher lesion count among chewers compared to nonchewers (IRR = 1.504, *p*  < 0.001). This association remained statistically significant even after adjusting for relevant clinical and demographic factors, with the fully adjusted model indicating a 29% higher expected number of lesions among BN chewers (IRR = 1.290, *p* = 0.034). These findings suggest that BN chewing was associated with PA pathology, reinforcing its negative impact beyond the periodontal tissues and implicating its role in endodontic disease.

Furthermore, a clear dose‐dependent association, wherein participants who chewed BN more frequently or over a longer duration, exhibited a higher number of PA lesions. Specifically, each additional packet chewed per day increased the likelihood of PA lesions by ~11%, while each additional year of BN use increased the likelihood by around 3%–4%. Although the frequency association lost statistical significance in the fully adjusted model, the duration of chewing remained a robust predictor of PA lesions across all models. These findings suggest a cumulative and chronic effect of BN exposure on PA tissues, consistent with the literature describing increased risk of pulpal and PA inflammation with prolonged masticatory trauma [36]. This highlights the importance of considering both the intensity and chronicity of BN chewing habits and association with endodontic disease risk.

It has been proposed that one of the primary reasons for the failure of endodontically treated teeth is coronal leakage resulting from the inadequacy of coronal restoration [37]. Song et al. [38] observed that 43.7% of teeth with adequate root canal fillings and poor restorations presented with AP. The success of root canal‐treated teeth appears to be dependent on the quality of the restoration and its hermetic seal. Therefore, it has been recommended that the final restoration be performed as soon as possible after successful root canal therapy [39]. In the current study, a significant relationship was found between inadequate indirect restorations/prosthesis and PA lesions (*p* < 0.05). This finding strengthens the evidence of the importance of ensuring an adequate coronal restoration to reduce the risk of endodontic failure.

OPGs are particularly advantageous in observational studies as they provide a comprehensive and relatively noninvasive view of the dentition, providing standardized images in a cost‐effective manner [40]. A recent systematic review found the diagnostic accuracy of AP on PA radiographs to be only around 4% higher than that of an OPG [41]. Furthermore, no significant difference was found between the two imaging modalities regarding the size of the AP lesion [42]; therefore, it proves to be a viable tool in such observational studies. The detection of PA lesions on panoramic radiographs becomes more accurate if observers are calibrated prior to the observation, as performed in the current study [43]. Also, a higher level of precision has been observed when using cone‐beam computed tomography (CBCT) imaging to detect AP [44]. Addressing the limitations of OPGs and PA radiographs, such as the superimposition of anatomical structures and the presence of dense cortical plates, can be effectively accomplished through the utilization of CBCT imaging [44]. Nevertheless, achieving higher accuracy through CBCT is accompanied by an increased dosage of radiation, which raises concerns regarding radiation exposure, requiring a careful review of the as low as reasonably achievable (ALARA) principles [45]. Centers involved in the current study use OPGs as a standard protocol for any patient attending the university clinic. The already established protocol was particularly advantageous as no additional costs or radiation exposure was incurred only for research purposes. Thus, radiographic evaluation could be performed without further radiographic interventions, which should be a fundamental principle when performing such in vivo studies [46].

The present study draws its sample from two distinct cities located in different Indian states, which inherently limits the generalizability of the findings. The prevalence of BN consumption varies significantly across India due to cultural, socioeconomic, and regulatory factors. For instance, ~12% of the population in Maharashtra are regular BN users, whereas the prevalence in Jammu and Kashmir is markedly lower, estimated at just 0.1% [4]. Such disparities complicate direct comparisons between the two populations and underscore the need for caution when interpreting aggregated findings. By including data from a moderate‐prevalence region (Maharashtra) and a low‐prevalence region (Jammu and Kashmir), our study contributes to a more nuanced understanding of these associations. It emphasizes the need for future multicentric studies that stratify risk based on regional consumption patterns and cultural practices. Additionally, this study did not collect data on cracked or fractured teeth, which are known consequences of BN chewing and may mediate the relationship between chewing habits and apical pathology. The study also did not include blood tests for assessments, nor did it collect data on heart/kidney diseases, which may affect the results and is a limitation of the study.

Limitations include the study’s cross‐sectional design, which precludes the assessment of temporality. AP was assessed radiographically on panoramic images only, without adjunctive clinical tests such as pulp sensibility testing or percussion, which may lead to misclassification of the endodontic status. Additionally, several potential AP‐related confounders were not measured, including fluoride exposure, dietary sugar intake, tooth fractures or cracks, and detailed socioeconomic indicators, which may influence susceptibility to pulpal infection and PA disease [47]. In BN chewers, these factors may interact in complex ways: Frequent consumption of sweetened betel quid or sugar‐rich foods increases caries risk, while cariogenic carbohydrates are a key driver of dental decay and its pulpal complications [48, 49]. At the same time, betel quid chewing is associated with severe tooth wear, periodontal breakdown, and tooth loss, which may predispose to cracks, structural compromise, and plaque‐retentive surfaces that favor pulpal and PA pathology [16, 50–52]. Socioeconomic disadvantage, which is common in many betel‐chewing communities, is itself linked with higher caries burden, limited access to dental care, and unhealthy dietary patterns, further compounding the risk profile for PA disease [49]. Because these caries‐related, structural, and dietary factors were not captured in the present analysis, residual confounding is likely, particularly when interpreting associations between BN chewing, pulpal infection, and AP.

BN consumers had more teeth with radiographic AP than nonchewers. This association may help clinicians consider BN chewing as a behavioral marker linked with a greater endodontic disease burden in populations where chewing is common. Prospective studies are needed to establish temporality, assess mechanisms, and evaluate the influence of unmeasured confounders such as diet, fluoride exposure, socioeconomic factors, and tooth fractures/cracks.

## 5. Conclusions

BN consumers presented a higher number of teeth with AP than nonchewers. In addition, a longer duration of BN chewing was significantly associated with the number of PA lesions than chewing frequency, suggesting a cumulative exposure effect. The study supports the aim of isolating the independent effect of BN without tobacco on the relationship with AP and highlights the importance of prevention and early clinical intervention in populations with high BN use. However, as a cross‐sectional study, it can only indicate a possible association rather than causation, and further longitudinal studies that consider additional confounding factors are needed to clarify and strengthen this relationship.

## Author Contributions

**Tousif Iqbal Nathani:** conceptualization, methodology, formal analysis, writing – original draft. **Marc Encinas**: conceptualization, methodology, writing – original draft, supervision. **Fernando Durán-Sindreu**: conceptualization, methodology, supervision. **Juan Gonzalo Olivieri**: conceptualization, supervision, project administration. **Vaibhav Kumar and Satvinder Singh**: investigation, data curation. **Shishir Singh**: investigation, data curation, formal analysis. All authors: writing – review and editing.

## Funding

No funding was received for this research.

## Ethics Statement

This study was approved by the institutional ethics committees of the participating centers (study codes: IEC/GDC32/20 and TDC/EC/17/2021). Written informed consent was obtained from all participants prior to enrollment.

## Conflicts of Interest

The authors declare no conflicts of interest.

## Supporting Information

Additional supporting information can be found online in the Supporting Information section.

## Supporting information


**Supporting Information** The STROBE checklist has been provided as a supporting document.

## Data Availability

The data that support the findings of this study are available from the corresponding author upon reasonable request.
