# Supplementary material for: The Potential Association Between Betel Nut Consumption and Apical Periodontitis: A Multicenter Cross‐Sectional Study
Source: Int J Dent. 2026 Jun 19;2026:2691950. doi: 10.1155/ijod/2691950 (PMC13280806; doi:10.1155/ijod/2691950)
Supplement: Supplementary file 1 — Supporting Information The STROBE checklist has been provided as a supporting document. [file IJOD-2026-2691950-s001.docx]

**STROBE Statement—Checklist of items that should be included in reports of cross-sectional studies**

This checklist has been completed with reference to the revised manuscript titled “The Potential Association Between Betel Nut Consumption and Apical Periodontitis: A Multicentre Cross-Sectional Study”.

Where an item was not applicable, this is indicated in the ‘Reported on page No./Location’ column. Locations refer to manuscript sections and table numbers.

| **Item No.** | **Recommendation** | **Reported on page No./Location** |
| --- | --- | --- |
| 1 | Title and abstract | Title page; Abstract |
| 2 | Background/rationale | Introduction |
| 3 | Objectives | Introduction (final paragraph) |
| 4 | Study design | Materials and Methods: Study design and setting |
| 5 | Setting | Materials and Methods: Study design and setting |
| 6 | Participants | Materials and Methods: Case selection; Inclusion and exclusion criteria |
| 7 | Variables | Materials and Methods: Variables and measurements |
| 8 | Data sources/measurement | Materials and Methods: Variables and measurements; Intraoral and radiographic examination |
| 9 | Bias | Discussion: Limitations |
| 10 | Study size | Materials and Methods: Sample size calculation |
| 11 | Quantitative variables | Materials and Methods: Data and statistical analysis |
| 12 | Statistical methods | Materials and Methods: Data and statistical analysis; Tables 2–4 |
| 13 | Participants (numbers) | Results; Table 1; Table 5 |
| 14 | Descriptive data | Table 1; Table 5; Table 6 |
| 15 | Outcome data | Table 1; Tables 2–4 |
| 16 | Main results | Results; Tables 2–4 |
| 17 | Other analyses | Results: correlation analysis; centre comparison (Table 5); BN chewer characteristics by centre (Table 6) |
| 18 | Key results | Discussion; Conclusions |
| 19 | Limitations | Discussion: Limitations |
| 20 | Interpretation | Discussion |
| 21 | Generalisability | Discussion: Generalisability paragraph |
| 22 | Funding | Declarations: Funding |
